# Counteracting Ageism and Fostering Lifelong Engagement via Multi-Generational School Music Opportunities

**DOI:** 10.1093/geroni/igaf122.708

**Published:** 2025-12-31

**Authors:** Lisa Lehmberg

**Affiliations:** University of Massachusetts Amherst, Amherst, Massachusetts, United States

## Abstract

Research has consistently shown that ageism—a socially constructed form of discrimination—negatively impacts the self-esteem and overall well-being of older adults worldwide, including in the United States. However, participation in musical activities has been shown to counteract these effects by enhancing self-esteem, fostering social belonging, and instilling a sense of purpose in older adults. While these benefits are well-documented, a critical question remains: How can younger individuals be encouraged to experience the benefits of music participation as they age? Through the lens of the strategies to reduce ageism shared in the World Health Organization’s Global Report on Ageism (2021), this presentation synthesizes research findings related to integrating older adults in U.S. school music settings, highlighting approaches for (a) fostering meaningful music connections between older adults and school music students, and (b) cultivating an early mindset of “lifelong musician in training.” In particular, multi-generational school music activities are recommended as potentially valuable opportunities to promote lifelong musical participation while challenging age-related stereotypes and enhancing the well-being of older adults.

